# Characteristic oscillatory brain networks for predicting patients with chronic migraine

**DOI:** 10.1186/s10194-023-01677-z

**Published:** 2023-10-18

**Authors:** Fu-Jung Hsiao, Wei-Ta Chen, Yu-Te Wu, Li-Ling Hope Pan, Yen-Feng Wang, Shih-Pin Chen, Kuan-Lin Lai, Gianluca Coppola, Shuu-Jiun Wang

**Affiliations:** 1 Brain Research Center, National Yang Ming Chiao Tung University, Taipei, Taiwan; 2 School of Medicine, National Yang Ming Chiao Tung University, Taipei, Taiwan; 3 Department of Neurology, Neurological Institute, Taipei Veterans General Hospital, 201, Shih Pai Rd Sec 2, Taipei, 11217 Taiwan; 4 Department of Neurology, Keelung Hospital, Ministry of Health and Welfare, Keelung, Taiwan; 5 Department of Medico-Surgical Sciences and Biotechnologies, Sapienza University of Rome, Polo Pontino, Latina, Italy

**Keywords:** Chronic migraine, Resting-state oscillatory connectivity, Pain-related network, Default mode network, Magnetoencephalography, Machine learning

## Abstract

To determine specific resting-state network patterns underlying alterations in chronic migraine, we employed oscillatory connectivity and machine learning techniques to distinguish patients with chronic migraine from healthy controls and patients with other pain disorders. This cross-sectional study included 350 participants (70 healthy controls, 100 patients with chronic migraine, 40 patients with chronic migraine with comorbid fibromyalgia, 35 patients with fibromyalgia, 30 patients with chronic tension-type headache, and 75 patients with episodic migraine). We collected resting-state magnetoencephalographic data for analysis. Source-based oscillatory connectivity within each network, including the pain-related network, default mode network, sensorimotor network, visual network, and insula to default mode network, was examined to determine intrinsic connectivity across a frequency range of 1–40 Hz. Features were extracted to establish and validate classification models constructed using machine learning algorithms. The findings indicated that oscillatory connectivity revealed brain network abnormalities in patients with chronic migraine compared with healthy controls, and that oscillatory connectivity exhibited distinct patterns between various pain disorders. After the incorporation of network features, the best classification model demonstrated excellent performance in distinguishing patients with chronic migraine from healthy controls, achieving high accuracy on both training and testing datasets (accuracy > 92.6% and area under the curve > 0.93). Moreover, in validation tests, classification models exhibited high accuracy in discriminating patients with chronic migraine from all other groups of patients (accuracy > 75.7% and area under the curve > 0.8). In conclusion, oscillatory synchrony within the pain-related network and default mode network corresponded to altered neurophysiological processes in patients with chronic migraine. Thus, these networks can serve as pivotal signatures in the model for identifying patients with chronic migraine, providing reliable and generalisable results. This approach may facilitate the objective and individualised diagnosis of migraine.

## Introduction

Migraine, a highly prevalent neurological disorder, is a disabling disease that affects over one billion individuals globally, with a global age-standardised prevalence of 14.4% [1]. This condition is characterised by recurrent headache accompanied by nausea, vomiting, photophobia, or phonophobia. Patients with chronic migraine (CM) experience substantial socioeconomic challenges and functional impairments [2]. Migraine is considered a prototypic functional disorder because of the absence of interictal symptoms and overt brain lesions. Thus, diagnostic uncertainty can lead to unnecessary medical tests and suboptimal treatment approaches.

With a strong genetic contribution, migraine is a complex brain network disorder caused by widespread structural and functional abnormalities in the brain regions responsible for multisensory, affective, and cognitive processing [3–8]. Thus, altered intrinsic brain connectivity has been observed in patients with migraine or pain disorders within (or among) the pain-related network (PN), default mode network (DMN), sensorimotor network (SMN), visual network (VN), and insula to DMN (Ins-DMN) [5, 9–16]. These observations indicate the vital role of network-based dysfunction in pathophysiological mechanisms. However, the debate regarding whether this evidence can definitively distinguish patients with migraine from those without migraine or those with other pain disorders is ongoing. Moreover, although aberrant brain patterns have been identified, neurologists still rely on traditional diagnostic tools for CM. This is primarily because most studies have demonstrated differences between groups, whereas clinicians need to make individualised treatment decisions. To harness the potential of electrophysiological signatures for individualised migraine diagnosis, the supervised machine learning (ML) approach is a promising technique. This approach involves developing algorithms and techniques that automatically identify patterns in data and utilise them to predict or classify future data. The combination of the network-based analysis of functional brain connectivity and ML approaches can achieve individualised diagnosis and treatment for CM, making the combination suitable for use in routine clinical practice.

In this study, we employed magnetoencephalography (MEG) to directly record neural activity across a wide frequency range and to analyse resting-state oscillatory connectivity within cortical networks. Compared with conventional scalp electroencephalography and functional MRI, MEG offers superior localisation accuracy and a more detailed understanding of the temporo-spectral dynamics of cortical activities, respectively [17]. To identify networks featuring electrophysiological alterations in CM, we analysed resting-state functional connectivity (FC) to determine the characteristic networks, including the PN, DMN, SMN, VN, and insula–DMN, underlying pathophysiological processes. In addition, we employed an ML-derived classification model to distinguish between patients with CM and healthy controls (HCs). Furthermore, we validated this model by using a new testing dataset and examined its efficacy on other datasets containing data on various chronic pain disorders, such as CM with comorbid fibromyalgia (CMFM), fibromyalgia (FM), and chronic tension-type headache (CTTH), and another migraine spectrum disorder (episodic migraine [EM]) to evaluate the generalisability of the model.

## Materials and methods

### Participants

All participants were aged between 20 and 60 years, were right-handed, had no history of systemic or major neurological diseases, had normal physical and neurological examination findings, and were enrolled from the headache clinic of Taipei Veterans General Hospital. HCs did not have personal or family histories of pain disorders and had not experienced any substantial pain condition in the previous year. Participants receiving prophylactic drugs, hormones, or other medications on a daily basis were excluded. This study was approved by the Institutional Review Board of Taipei Veterans General Hospital (VGHTPE: IRB 2015-10-001BC), and all participants provided written informed consent before study commencement.

Patients were diagnosed as having EM or CM in accordance with *International Classification of Headache Disorders, Third Edition* (*ICHD-3*) [18]. These patients had not undergone preventive migraine treatment and denied overusing headache medications. FM was diagnosed in accordance with the modified 2010 criteria of American College of Rheumatology [19], and patients with FM did not have any autoimmune rheumatic disease. Patients with CMFM were included if they met the aforementioned criteria for CM and FM. Patients were diagnosed as having CTTH in accordance with the stringent ICHD-3 criteria. These patients were required to meet all the following 4 headache characteristics that are defined as the core syndromes of TTH: bilateral, mild-to-moderate intensity, nonpulsating, and not aggravated by routine physical activity. Moreover, they were required to report no migrainous features (nausea, vomiting, photophobia, or phonophobia) associated with their headache, although the original criteria included the presence of either photophobia or phonophobia.

### Study design

All participants were administered semi-structured questionnaires to collect their demographic data, and they completed psychometric evaluations, such as the Hospital Anxiety and Depression Scale (HADS) [20]. The headache profile of patients with migraine was recorded, including the number of headache days per month, duration of headache attacks (in months), and average headache intensity in the previous year. In addition, the Migraine Disability Assessment (MIDAS) questionnaire was administered to assess the extent of migraine-related disability [21]. Throughout the study, all patients maintained a headache diary in which they recorded the date and time of headache attacks, pain intensity, associated symptoms, medication usage (if any), and menstrual periods.

The FM profile of patients with FM was recorded, including the duration of widespread musculoskeletal pain attacks (in months), the extent of pain distribution (the widespread pain index), accompanying somatic or psychiatric symptoms (symptom severity scale) [19], and the frequency of painkiller usage per month. In addition, these patients were administered the Revised Fibromyalgia Impact Questionnaire to evaluate their FM-associated functional disability.

The CTTH profile of patients with CTTH was recorded, including the number of headache days per month, duration of headache attacks (in months), average headache intensity over the previous year, and frequency of painkiller usage per month.

Each participant underwent MEG recording. For patients with headaches, the recording took place during the interictal period, which was defined as the absence of an acute migraine attack within the 2 days before and after the MEG recording. Patients with CM and CMFM could have a background or interval headache during this period [5]. However, if an acute attack occurred or if analgesics, triptans, or ergots were used within 48 h before recording, the MEG session was rescheduled. The timing relationship between MEG recordings and headache episodes was determined either from information provided in the headache diary or through follow-up phone calls.

### MEG recording

A whole-scalp 306-channel MEG system (Vectorview; Elekta Neuromag, Helsinki, Finland) was used to record brain activity. To ensure accurate head positioning, four coils were placed on participants’ scalp in alignment with the head coordinate frame based on Cartesian coordinates with respect to the nasion and two preauricular points. The positions of these coils were mapped using a three-dimensional (3D) digitiser. For precise registration, approximately 100 additional scalp points were digitised. These landmarks facilitated alignment between MEG and MRI coordinate systems for each participant. Subsequently, individual brain T1 images were obtained using a 3-T MRI system (Discovery 750; GE Medical Systems, WI, USA) with the following parameters: repetition time: 9.4 ms, echo time: 4 ms, recording matrix: 256 × 256 pixels, field of view: 256 mm, and slice thickness: 1 mm.

During the 5-minute resting-state MEG recording, participants were instructed to close their eyes; remain awake, relax; and avoid any specific tasks. The digitisation rate for recordings was set at 600 Hz. If participants fell asleep or exhibited excessive head movement during recording, the session was paused and conducted again. To facilitate offline artifact elimination, the simultaneous recordings of electrooculography (EOG) and electrocardiography (ECG) activities were obtained. To account for sensor and environmental noise, a 3-minute empty-room recording was performed. This recording was used to calculate noise covariance for offline source analysis.

### MEG data preprocessing and analysis

**Fig. 1:**
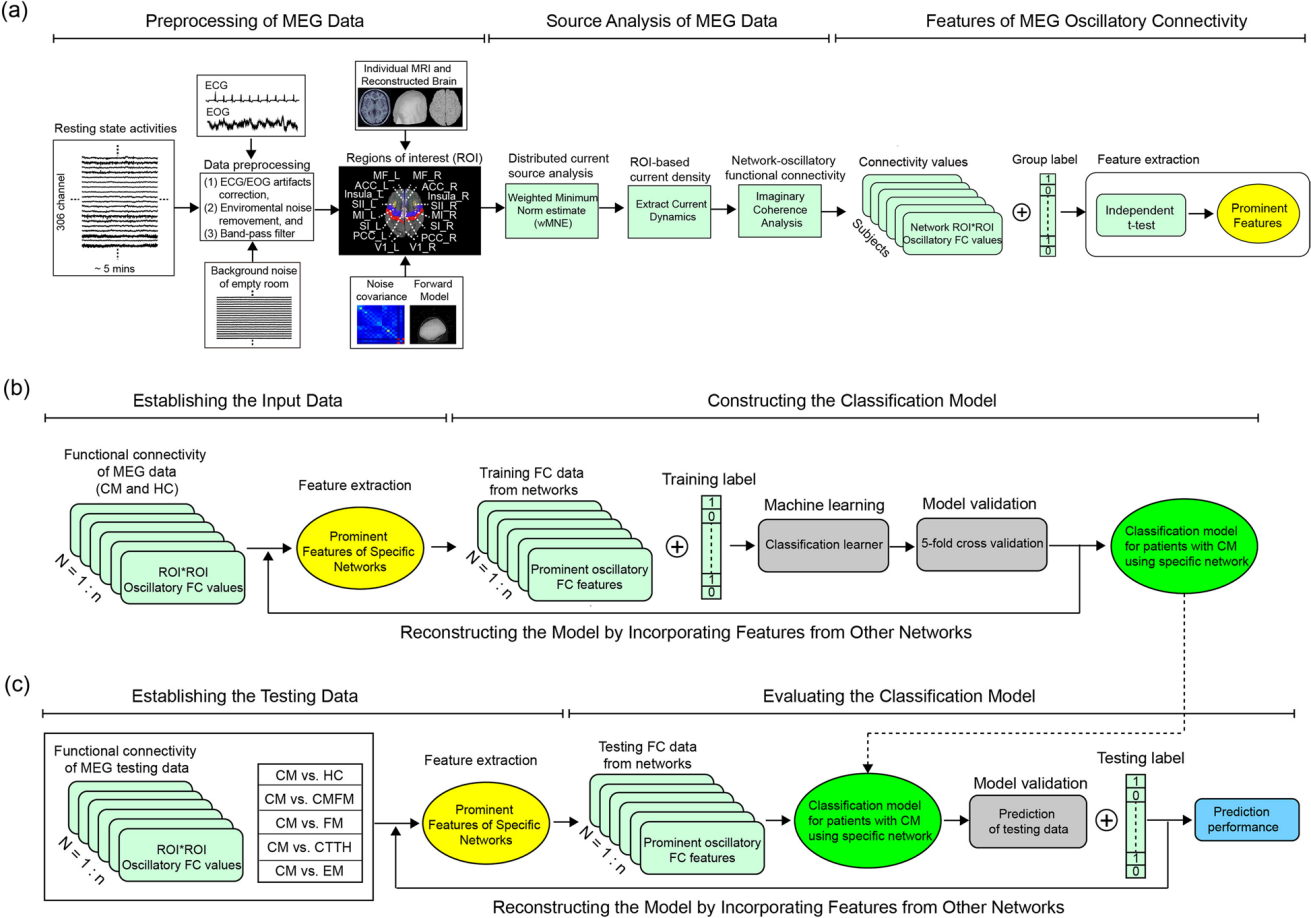
Procedure of data analysis. Pipeline of resting-state MEG preprocessing, oscillatory connectivity, and machine learning analysis. ECG, electrocardiogram; EOG, electrooculogram; FC, functional connectivity; ROI, regions of interest; HC, healthy controls; CM, chronic migraine; FM, fibromyalgia; CMFM, CM with comorbid FM; CTTH, chronic tension-type headache; EM, episodic migraine; MF, media frontal; ACC, anterior cingulate cortex; SII, secondary somatosensory; MI, primary motor; SI, primary somatosensory; PCC, posterior cingulate cortex; V1, primary visual; L, left; R, right

To mitigate the effect of nonbrain or environmental artifacts in spontaneous resting-state MEG data (Fig. 1), we implemented several measures. First, segments containing artifacts resulting from environmental noise were discarded. Second, notch filters were employed to eliminate contamination at 60 Hz and its harmonics, which are associated with powerline interference. Finally, identified events related to heartbeat and eye blinking, as obtained from ECG and EOG data, respectively, were used to create separate projections through principal component analysis; these events were selectively eliminated from data [22].

T1-weighted brain images were employed to create a cortical model for source analysis, and these images underwent automatic reconstruction into a surface model by using BrainVISA (version 4.5.0, http://brainvisa.info). Subsequently, the anatomical magnetic resonance images and reconstructed cortical surface were coregistered with the corresponding MEG dataset.

To establish a distributed source model of MEG data for indicating source-based cortical activation, we used Brainstorm [23]. The overlapping sphere method was employed for forward modelling [24], and an inverse operator was calculated through a depth-weighted minimum norm estimate (MNE) analysis. In this source model, each grid point (vertex) on the cortical surface represented a current dipole. To facilitate group analysis, the cortical activation of each participant was morphed into a common source space. MNE parameters used in this study are consistent with those described in our previous studies [4, 7, 25]. In this study, regions of interest (ROIs) were defined in the structural T1 template volume by using Mindboggle cortical parcellation [26]. The following ROIs were selected for studying networks: (1) PN: bilateral primary motor (MI), primary somatosensory (SI), secondary somatosensory (SII), anterior cingulate cortex (ACC), and insula; (2) DMN: bilateral medial frontal, inferior parietal, posterior cingulate cortex, lateral temporal, and medial temporal cortex; (3) SMN: bilateral SI, SII, and MI; (4) VN: bilateral primary visual cortex; and (5) Ins-DMN: insula and DMN. The averaged current density from all vertices in each identified ROI was calculated for the subsequent analysis of FC within each network.

Oscillatory FC between the ROIs in each network was computed on the basis of the dynamic current intensity by using the imaginary coherence method, and it was categorised into the following frequency bands: delta (1–4 Hz), theta (5–7 Hz), alpha (8–13 Hz), beta (14–25 Hz), and gamma (26–40 Hz). This method measures the coupling of the oscillatory phases of activation dynamics between two cortical sources while minimising crosstalk effects between sources [27]. FC was calculated at a frequency resolution of 0.586 Hz [5]. In this study, the node strength for each ROI within each network, which was defined as the sum of FC values for the node being examined (ROI), was individually estimated to represent the magnitude of FC in each frequency band in each network.

### Establishing the classification model

Feature selection is a crucial step for enhancing classification performance and reducing computational complexity. This step involves selecting a subset of relevant features from the original set. In this study, we performed univariate analysis (independent *t* test) with false detection of discovery (FDR) correction to identify discriminative features from each network based on the group factor (HC vs. CM). These discriminative features extracted from distinct networks were subsequently used to construct training and testing datasets (Fig. 1). In this study, classification models constructed from the combinations of prominent networks were examined. The PN represented altered intrinsic connectivity for migraine and was associated with chronification [5].

In this study, a classification model was established using the ML toolbox of MATLAB software (R2019a). Machine learning algorithms transform input feature vectors into a high-dimensional space, enabling the creation of a linear classification system. By implementing algorithms by using training data, an optimal hyperplane that minimises risks and generates a classification model can be determined. The supervised learning approach was employed to train classifiers, including decision trees, discriminant analysis, naïve Bayes classifiers, support vector machine (SVM), and k-nearest neighbour. These classifiers decoded two conditions (CM vs. HC) in a pairwise manner. To mitigate overfitting, the models were trained using a 5-fold leave-one-out cross-validation technique. The performance of each classification model was evaluated by examining its accuracy, sensitivity, specificity, and area under the curve (AUC) values. In the classification model, Shapley values were computed to assign a value to each feature, revealing their contribution to a particular prediction [28]. The averaged Shapley values across the classification models with satisfactory performance were calculated to determine the importance of each feature in identifying patients with CM.

After establishing the classification models, we validated them to determine their generalisability across various testing datasets, including new testing datasets (CM vs. HC) and other datasets (CM vs. CMFM, CM vs. FM, CM vs. CTTH, and CM vs. EM; Fig. 1). Features in these testing datasets were selected on the basis of the discriminative feature index. The testing dataset labels were blinded, and the classification models were applied to discriminative features without any training. The classification accuracy and AUC values were obtained for each model. In addition, to examine the statistical significance of the predictive accuracy, we employed nonparametric permutation tests (10,000 iterations). These tests involved estimating the statistics of the classification accuracy by permuting labels. Subsequently, we determined the proportion of permutations in this null distribution that achieved higher accuracy than the true labels and then divided it by the total number of permutations. This calculation provides an estimate of the significance of the accuracy relative to chance.

## Results

### Demographic and clinical data

This study included 350 participants—70 HCs, 100 patients with CM, 40 patients with CMFM, 35 patients with FM, 30 patients with CTTH, and 75 patients with EM. Of them, the data of 56 HCs and 80 patients with CM were included in the training dataset. Table 1 provides a summary of the demographic and clinical characteristics of all the participants in the training dataset. The two groups in the training dataset did not significantly differ in terms of age or sex. Anxiety (HADS_A) and depression (HADS_D) scores were higher in the CM group than in the HC group (HADS_A, *p* < 0.001; HADS_D, *p* < 0.001). The testing dataset consisted of the data of 14 HCs, 20 patients with CMs, 40 patients with CMFM, 35 patients with FM, 30 patients with CTTH, and 75 patients with EM (Table 2). The groups in the testing dataset did not significantly differ in terms of age. However, the FM group included significantly more women than those in the CM, EM, and CTTH groups (CM, *p* = 0.02; EM, *p* < 0.001; CTTH, *p* < 0.001). Similar to the findings for the groups in the training dataset, anxiety and depression scores were lower in the HC group than in the other pain disorder groups (anxiety: CM, *p* < 0.001; CMFM, *p* < 0.001; EM, *p* < 0.001; FM, *p* < 0.001; CTTH, *p* = 0.001. depression: CM, *p* = 0.007; CMFM, *p* < 0.001; FM, *p* = 0.001; CTTH, *p* = 0.043). As expected, the patients with CM or CMFM had more monthly headache days than did those with EM (CM, *p* < 0.001; CMFM, *p* < 0.001). The CTTH group had lower headache severity in the last year than did the CM, CMFM, and EM groups (CM, *p* = 0.001; CMFM, *p* < 0.001; EM, *p* < 0.001). The MIDAS scores were higher in the patients with CM or CMFM than in those with EM (CM, *p* = 0.023; CMFM, *p* = 0.002). Notably, psychometric scores were comparable among the CM, CMFM, CTTH, FM, and EM groups.

**Table 1:** Demographics and clinical profiles of participants in the training dataset

	HC	CM
N	56	80
Demographics		
Age (years)	41.4 ± 8.3	39.4 ± 11.6
Sex	39 F/17 M	69 F/11 M
Psychometrics		
HADS_A	4.6 ± 3.5	8.4 ± 4.1^**^
HADS_D	3.9 ± 3.0	6.3 ± 3.9^*^
Migraine profile		
Headache days (/month)		20.1 ± 6.4
Disease duration (months)		198.6 ± 144.2
Severity of last year (0–10)		6.3 ± 2.1
MIDAS		45.7 ± 61.2

*HC *Healthy control, *CM *Chronic migraine, *F *Female, *M *Male, *HADS *Hospital anxiety and depression score, *A *Anxiety, *D *Depression, *BDI *Beck’s depression inventory; *MIDAS *Migraine disability assessment scores

*, *p* = 0.001; **, *p* < 0.001

**Table 2:** Demographics and clinical profiles of participants in the testing data sets

	HC	CM	CMFM	EM		FM		CTTH
N	14	20	40	75	N	35	N	30
Demographics					Demographics		Demographics	
**Age (years)**	38.3 ± 11.7	36.2 ± 10.5	40.5 ± 12.5	36.9 ± 10.6	**Age (years)**	42.0 ± 11.5	**Age (years)**	42.9 ± 12.9
**Sex**	11F/3M	15F/5M	35F/5M	59F/16M	**Sex**	34F/1M^ξ^	**Sex**	16F/14M
Psychometrics					Psychometrics		Psychometrics	
**HADS_A**	3.7 ± 2.5^#^	9.7 ± 5.4	10.2 ± 4.5	8.3 ± 3.9	**HADS_A**	10.3 ± 3.8^Ψ^	**HADS_A**	8.1 ± 3.7^ψ^
**HADS_D**	3.7 ± 2.9^$^	7.6 ± 4.3	8.9 ± 5.4	5.9 ± 3.4	**HADS_D**	8.1 ± 4.2^η^	**HADS_D**	6.4 ± 4.2^ζ^
Migraine profile					FM profile		CTTH profile	
**Headache days (/month)**	-	22.6 ± 6.2	22.1 ± 6.8	6.9 ± 4.9^&^	**WPI**	11.4 ± 4.9	**Headache days (/month)**	22.8 ± 6.2
**Disease duration (months)**	-	182.9 ± 157.5	236.4 ± 175.9	186.4 ± 106.6	**SSS**	7.1 ± 2.2	**Disease duration (months)**	161.8 ± 156.8
**Severity of last year (0-10)**	-	6.1 ± 2.2	6.8 ± 2.0	5.9 ± 2.0	**FIQR**	40.6 ± 18.0	**Severity of last year (0-10)**	4.2 ± 1.6^φ^
**MIDAS**	-	51.1 ± 74.9	55.9 ± 62.9	22.7 ± 26.6^Φ^	**Disease duration (months)**	226.3 ± 169.0	**Painkiller use (days/month)**	5.3 ± 10.1

*HC *Healthy control, *CM *Chronic migraine, *CMFM *Chronic migraine with comorbid fibromyalgia, *EM *Episodic migraine, *FM *Fibromyalgia, *CTTH *Chronic tension-type headache, *F *Female, *M *Male, *HADS *Hospital anxiety and depression score, *A *Anxiety, *D *Depression, *BDI *Beck’s depression inventory, *MIDAS *Migraine disability assessment scores, *WPI *Widespread pain index, *SSS *Symptom severity scale, *FIQR *Revised fibromyalgia impact questionnaire

#, smaller in HC (vs. CM, *p* < 0.001; vs. CMFM, *p* < 0.001; vs. EM, *p* < 0.001). $, smaller in HC (vs. CM, *p* = 0.007; vs. CMFM, *p* < 0.001). &, smaller in EM (vs. CM, *p* < 0.001; vs. CMFM, *p* < 0.001). Φ, smaller in EM (vs. CM, *p* = 0.023; vs. CMFM, *p* = 0.002). ξ, different from CM (*p* = 0.02), EM (*p* < 0.001) and CTTH (*p* < 0.001). Ψ, larger than HC (*p* < 0.001). η, larger than HC (*p* = 0.001). ψ, larger than HC (*p* = 0.001). ζ, larger than HC (*p* = 0.043). ϕ, smaller in CTTH (vs. CM, *p* = 0.001; vs. CMFM, *p* < 0.001; vs. EM, *p* < 0.001)

### Aberrant FC within distinct network in pain disorders

**Fig. 2:**
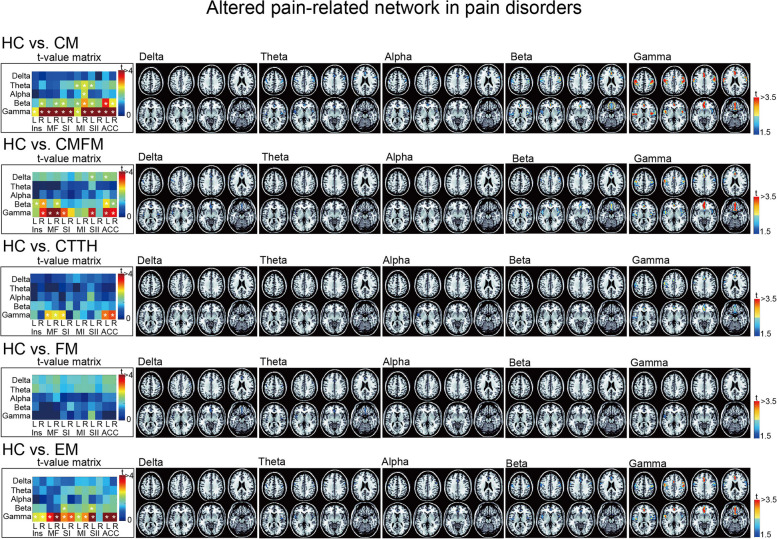
Aberrant pain-network connectivity in pain disorders. Differences in oscillatory connectivity within the pain-related network between patients with distinct pain disorders and healthy controls (HCs). The t-value matrix presents statistical results for groups with a spatial-oscillatory pattern. The t values are then mapped onto brain images in the axial view, with colours representing corresponding values. CM, chronic migraine; FM, fibromyalgia; CMFM, CM with comorbid FM; CTTH, chronic tension-type headache; EM, episodic migraine; L, left; R, right. Ins, insula; MF; medial frontal; SI, primary somatosensory; MI, primary motor; SII, secondary somatosensory; ACC, anterior cingulate cortex; L, left; R, right; *, corrected *p* < 0.05

FC measures revealed significant alterations in node strength within specific networks for the pain disorder groups compared with the HC group. In the PN, beta connectivity and gamma connectivity were decreased in the CM, CMFM, and EM groups (all *p* < 0.05 with FDR corrections and t-values represented using colour coding**)**. In the gamma band, a large portion of pain-related areas exhibited alterations. However, in the beta band, more areas (except the left SI and SII) were preserved in the EM group than in the CM and CMFM groups. Decreased connectivity in the theta and alpha bands was observed only in the CM group. Moreover, although the CTTH group exhibited decreased PN connectivity in the gamma band, the FM group exhibited intact FC. In general, in the PN, patients with CM presented widespread spatial and multifrequency deteriorations. These discriminative connections were illustrated by adjacency matrices spanning from the delta to gamma bands between the HC and pain disorder groups (left part of Fig. 2), and these features were topographically displayed on axial MRIs in different frequency bands (right part of Fig. 2).

**Fig. 3:**
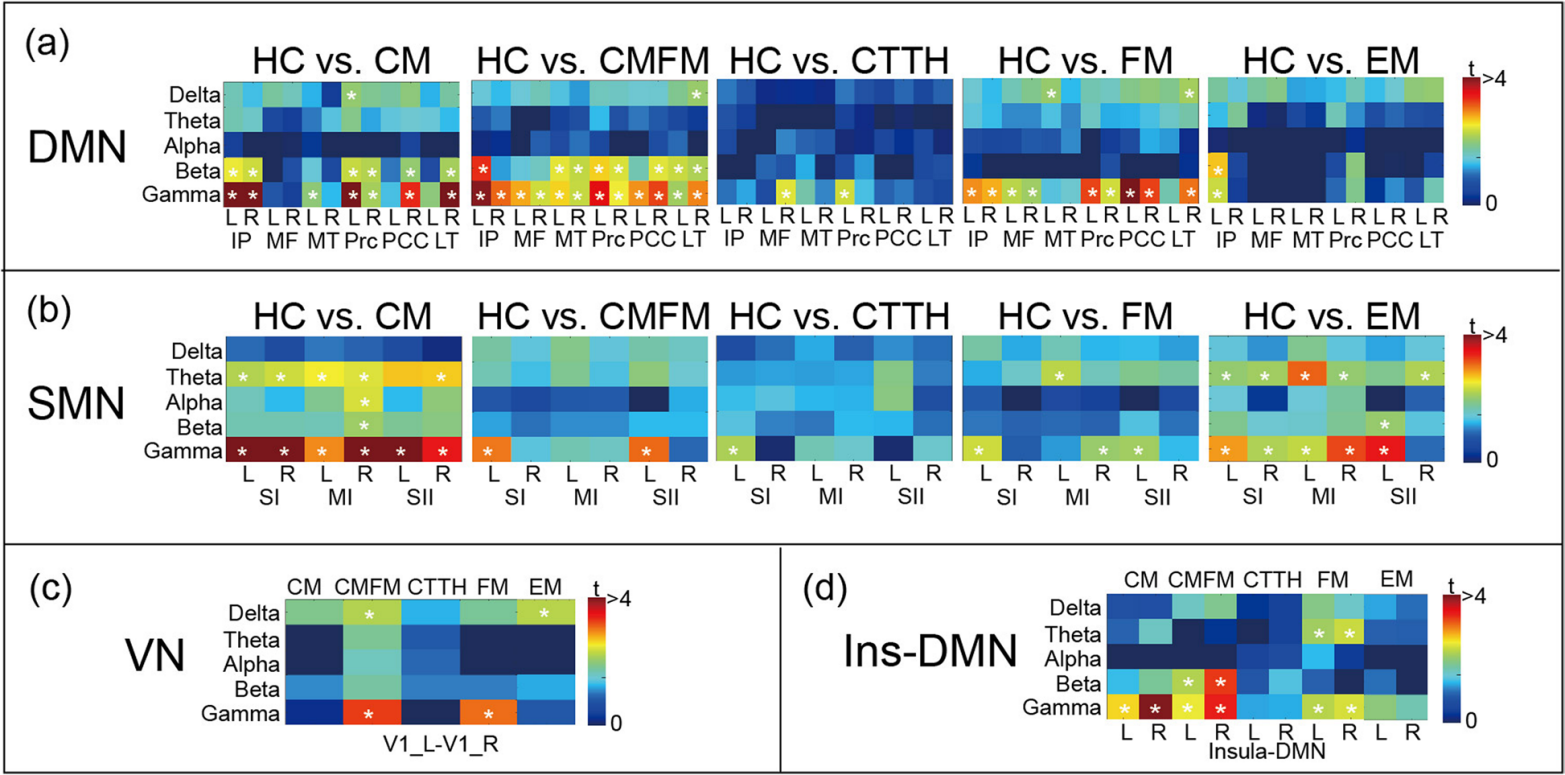
Altered network connectivity in pain disorders. Differences in oscillatory connectivity within the default mode network (DMN), sensorimotor network (SMN), visual network (VN), and insula to DMN network (Ins-DMN) between patients with distinct pain disorders and healthy controls (HCs). The t-value matrix presents statistical results for groups with a spatial-oscillatory pattern. CM, chronic migraine; FM, fibromyalgia; CMFM, CM with comorbid FM; CTTH, chronic tension-type headache; EM, episodic migraine; IP, inferior parietal; MF; medial frontal; MT, medial temporal; Prc, precuneus; PCC, posterior cingulate cortex; LT, lateral temporal; SI, primary somatosensory; MI, primary motor; SII, secondary somatosensory; V1, primary visual cortex; L, left; R, right; *, corrected *p* < 0.05

**Fig. 4:**
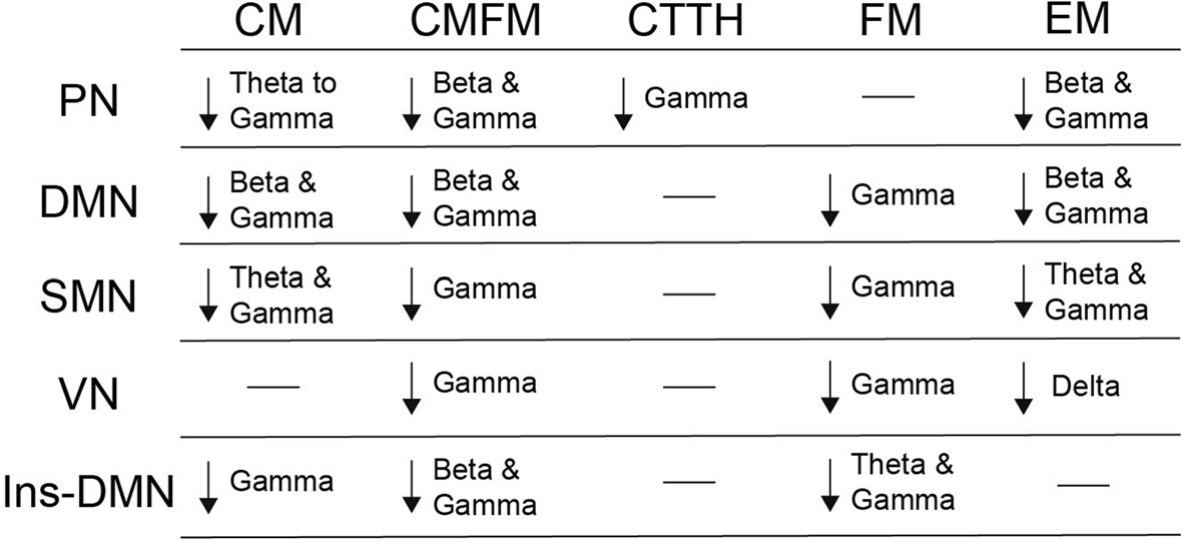
Summary of altered oscillatory connectivity within distinct networks in different pain disorders. CM, chronic migraine; FM, fibromyalgia; CMFM, CM with comorbid FM; CTTH, chronic tension-type headache; EM, episodic migraine; PN, pain-related network; DMN, default mode network; SMN, sensorimotor network; VN, visual network; Ins-DMN, insula to DMN

In the DMN (Fig. 3a), we noted a decrease in beta and gamma connectivity in the CM, CMFM, and EM groups (all corrected *p* < 0.05 with t-values represented using colour coding). However, the CMFM group exhibited the highest number of affected brain areas, followed by the CM and EM groups. In addition, decreased gamma band connectivity was observed in the CTTH and FM groups. The CTTH group exhibited only a few alterations only in the right medial frontal and left precuneus areas. In summary, the CM and CMFM groups exhibited a significant decline in DMN function, whereas the CTTH and EM groups exhibited minimal deviations in FC. In the SMN (Fig. 3b), we observed decreased theta and gamma connectivity in the SI, SII, and MI areas in the CM, FM, and EM groups (all corrected *p* < 0.05 with t-values represented using colour coding). Altered alpha connectivity and beta connectivity were noted in the right MI area in the CM group. Decreased connectivity was detected in few SMN areas in the CMFM and CTTH groups. However, the CM group exhibited abnormal connectivity between most SMN areas across various frequency bands. In the VN (Fig. 3c), the CMFM and FM groups exhibited decreased connectivity, whereas the CM and CTTH groups exhibited normal connectivity. Regarding Ins–DMN connectivity (Fig. 3d), alterations in connectivity in the beta and gamma frequency bands were observed in the CM, CMFM, and FM groups. Only the FM group exhibited a reduction in theta connectivity. Taken together, these findings indicate that each pain disorder has neuropathological mechanisms that might be characterised by aberrant network connectivity, as depicted in Fig. 4, from the perspectives of networks and frequency bands.

### Classification model using network connectivity for CM

By utilising discriminative features obtained from FC between the HC and CM groups (as mentioned in the earlier text), which included oscillatory connectivity at various frequencies within the PN, DMN, SMN, and Ins–DMN, we established training datasets for constructing classifiers. We examined the performance of different classification models based on the combinations of these prominent networks: (1) PN, DMN, SMN, and Ins–DMN; (2) PN, DMN, and SMN; (3) PN, DMN, and Ins–DMN; (4) PN, SMN, and Ins–DMN; (5) PN and DMN; (6) PN and SMN; (7) PN and Ins–DMN; (8) PN; (9) DMN; (10) SMN; and (11) Ins–DMN.

**Fig. 5:**
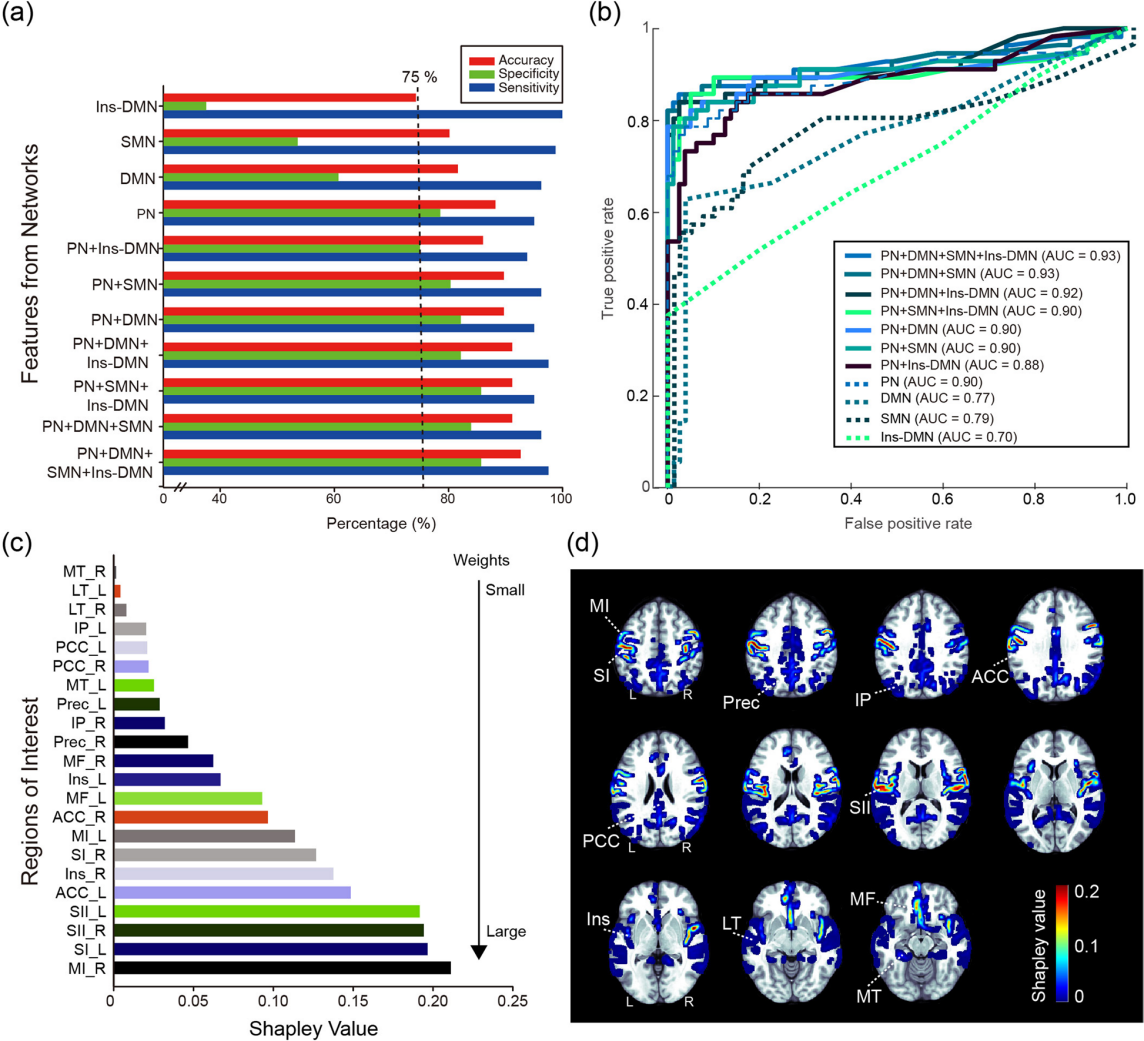
Performance of classification models with distinct network features for CM versus HC. **a** Comparisons of model performance with distinct features. **b** Receiver operating characteristic curves and area under the curve (AUC) values for different models. **c** Averaged Shapley values from prominent models for different brain areas. **d** Shapley values are then mapped onto axial-view brain images and colour-coded. PN, pain-related network; DMN, default mode network; SMN, sensorimotor network; VN, visual network; Ins-DMN, insula to DMN. MT, medial temporal; LT, lateral temporal; IP, inferior parietal; PCC, posterior cingulate cortex; Prec, precuneus; MF, media frontal; Ins, insula; ACC, anterior cingulate cortex; MI, primary motor; SI, primary somatosensory; SII, secondary somatosensory; L, left; R, right

The classification models exhibited varying accuracies, ranging from 74.3 to 92.6%, for differentiating between CM and HC in the training datasets (Fig. 5a and b) by using the discriminative features of the following networks: (1) PN, DMN, SMN, and Ins–DMN (SVM with median gaussian kernel; accuracy: 92.6%, sensitivity: 0.97, specificity: 0.86, AUC: 0.93); (2) PN, DMN, and SMN (SVM with median gaussian kernel; accuracy: 91.2%, sensitivity: 0.96, specificity: 0.84, AUC: 0.93); (3) PN, DMN, and Ins–DMN (SVM with median gaussian kernel; accuracy: 91.2%, sensitivity: 0.95, specificity: 0.85, AUC: 0.92); (4) PN, SMN, and Ins–DMN (SVM with linear kernel; accuracy: 91.2%, sensitivity: 0.97, specificity: 0.82, AUC: 0.9); (5) PN and DMN (SVM with median gaussian kernel; accuracy: 89.9%, sensitivity: 0.95, specificity: 0.82, AUC: 0.9); (6) PN and SMN (SVM with median gaussian kernel; accuracy: 89.7%, sensitivity: 0.96, specificity: 0.8, AUC: 0.9); (7) PN and Ins–DMN (SVM with median gaussian kernel; accuracy: 86.0%, sensitivity: 0.94, specificity: 0.75, AUC: 0.88); (8) PN (SVM with median gaussian kernel; accuracy: 88.2%, sensitivity: 0.95, specificity: 0.78, AUC: 0.9); (9) DMN (SVM with fine gaussian kernel; accuracy: 81.6%, sensitivity: 0.96, specificity: 0.61, AUC: 0.77), (10) SMN (SVM with median gaussian kernel; accuracy: 80.1%, sensitivity: 0.98, specificity: 0.53, AUC: 0.79), and (11) Ins–DMN (SVM with fine gaussian kernel; accuracy: 74.3%, sensitivity: 1.0, specificity: 0.37, AUC: 0.7). These results indicate the varying performance of different classification models based on the different combinations of prominent networks. Models with performance values below 0.75 were excluded from further validation, including those constructed from the DMN, SMN, and Ins–DMN alone. In addition, the averaged Shapley values for the eight classification models revealed the significance of each brain area within the networks for identifying patients with CM. Furthermore, these values were visually represented on axial MRIs (Fig. 5c and d). In particular, the connectivity of the MI, SI, SII, ACC, and insula areas was crucial for constructing a reliable classification model.

### Generalisability of the classification model

**Fig. 6:**
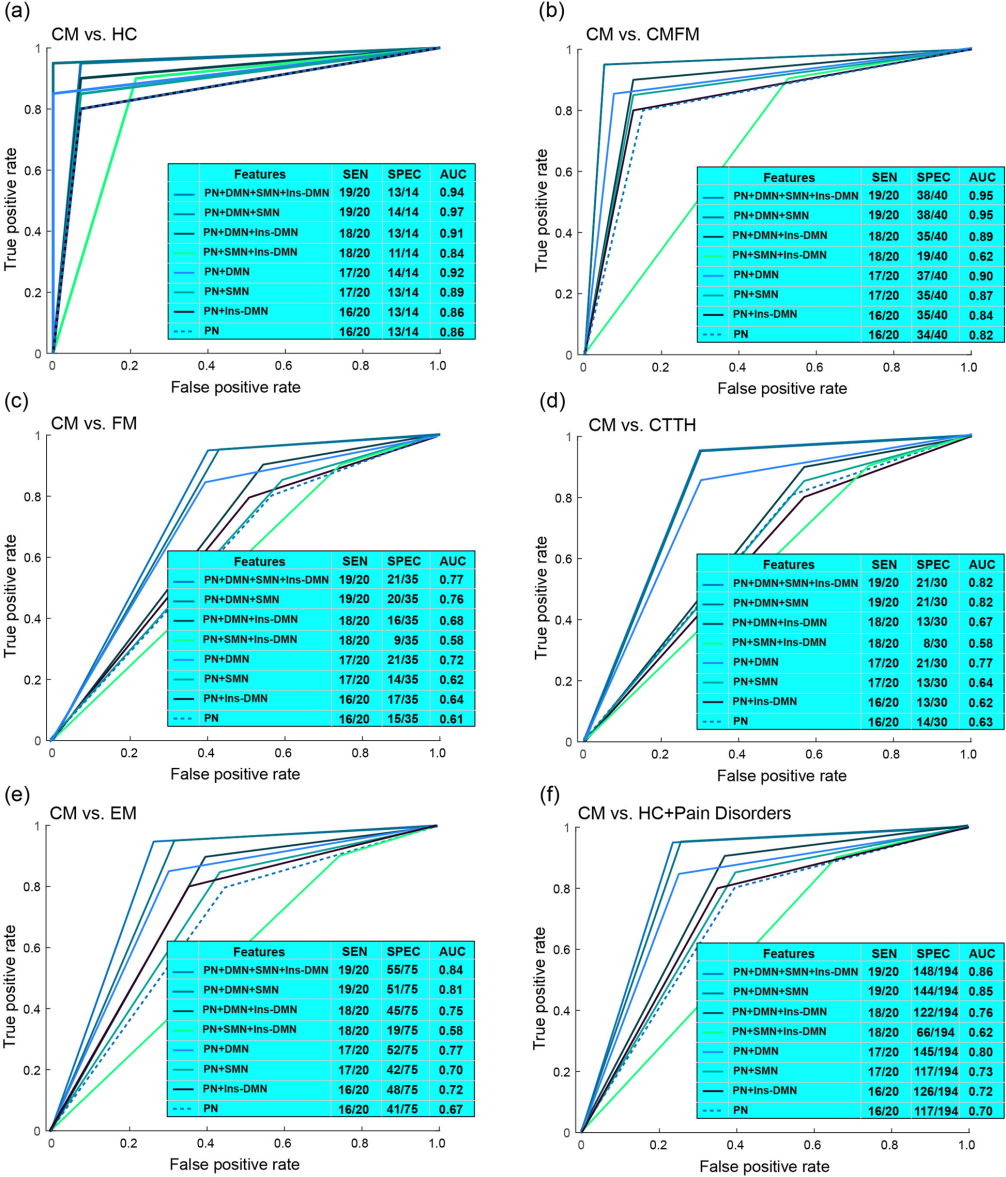
Validation of classification models for identifying patients with CM. Classification performance with distinct network features between (**a**) chronic migraine (CM) versus healthy controls (HCs), (**b**) CM versus CM with comorbid fibromyalgia (CMFM), (**c**) CM versus fibromyalgia (FM), (**d**) CM versus chronic tension-type headache (CTTH), (**e**) CM versus episodic migraine (EM). SEN, sensitivity; SPEC, specificity; AUC, the area under the curve. PN, pain-related network; DMN, default mode network; SMN, sensorimotor network; Ins–DMN, insula to DMN

**Fig. 7:**
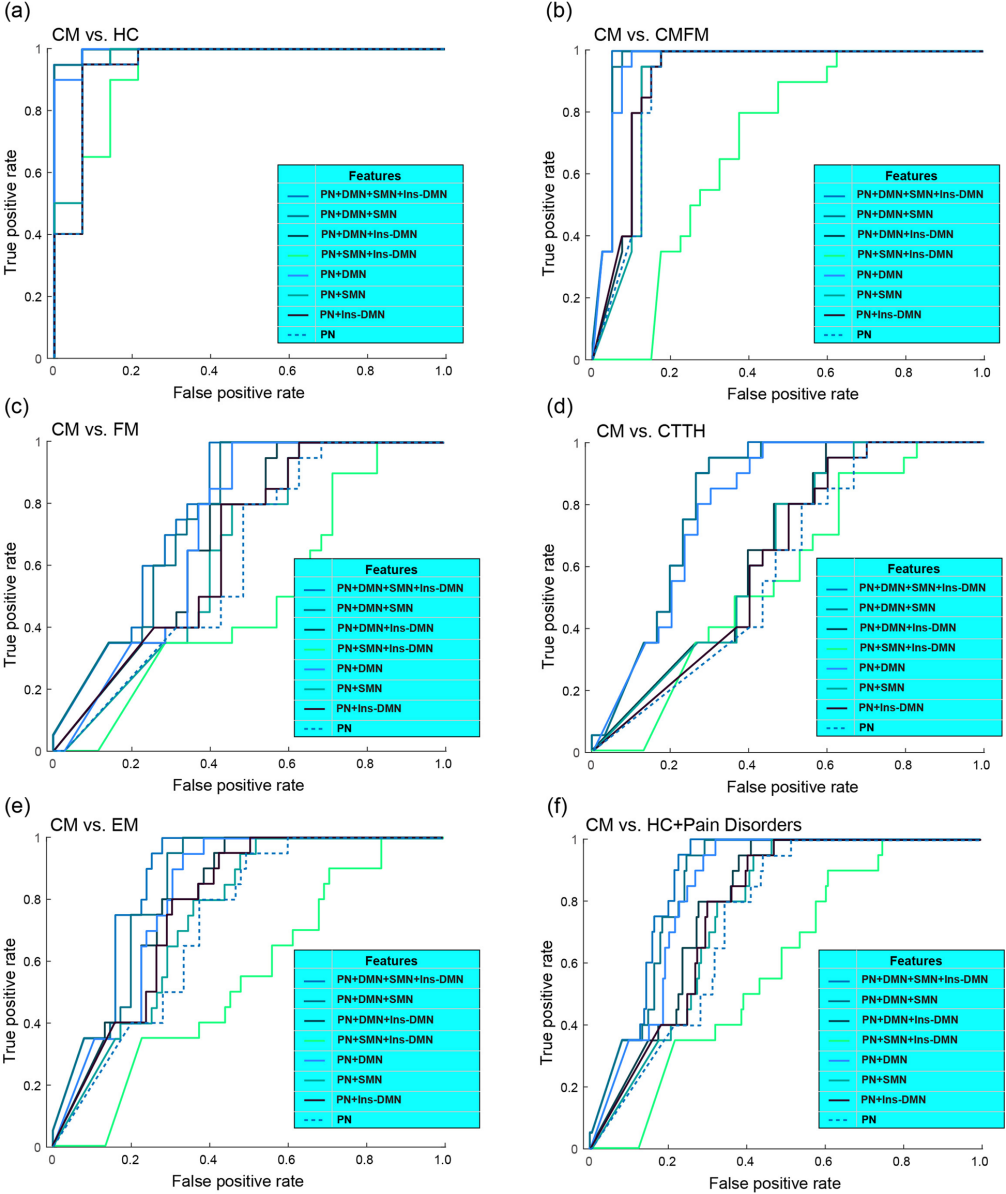
Receiver operating characteristic curves plotted using decisive values for distinct network features between (**a**) chronic migraine (CM) versus healthy controls (HCs), (**b**) CM versus CM with comorbid fibromyalgia (CMFM), (**c**) CM versus fibromyalgia (FM), (**d**) CM versus chronic tension-type headache (CTTH), (**e**) CM versus episodic migraine (EM). PN, pain-related network; DMN, default mode network; SMN, sensorimotor network; Ins–DMN, insula to DMN

To examine the generalisability of the eight classification models, we utilised an independent dataset containing the data of 20 patients with CM and 14 HCs. These models demonstrated high accuracies, ranging from 85.3 to 97.0% (all *p* < 0.0001), and excellent AUC values, ranging from 0.84 to 0.97 (Fig. 6a). In addition, we evaluated the performance of these models for distinguishing CM from other chronic pain disorders by using the following datasets. (1) The first dataset consisted of the data of 20 patients with CM and 40 patients with CMFM (Fig. 6b). The model yielded favourable results with accuracies ranging from 83.3 to 95.0% (all *p* < 0.0001) and AUCs ranging from 0.82 to 0.95. However, the model that incorporated features from the PN, SMN, and Ins–DMN exhibited a lower accuracy and AUC of 0.62. (2) The second dataset consisted of the data of 20 patients with CM and 35 patients with FM (Fig. 6c). Some models, specifically those incorporating features from (a) the PN, DMN, SMN, and Ins–DMN; (b) PN, DMN, and SMN; and (c) PN and DMN, displayed high accuracies (all *p* > 69.1% and *p* < 0.001) and AUC values (all *p* > 0.7). (3) The third dataset contained the data of 20 patients with CM and 30 patients with CTTH (Fig. 6d). Similar to the validations of CM versus FM, the three models exhibited high accuracies (all *p* > 76.0% and *p* < 0.0001) and AUC values (all *p* > 0.75). Moreover, the model performance for classifying different migraine subtypes was evaluated using a dataset comprising the data of 20 patients with CM and 75 patients with EM (Fig. 6e). With the exception of two models (one incorporating features from the PN, SMN, and Ins–DMN and another using PN features alone), both of which had AUC values below 0.7, all the other models exhibited high accuracies, ranging from 62.1 to 77.8% (all *p* < 0.001), and AUC values, ranging from 0.7 to 0.84. Finally, in terms of distinguishing the CM group from all other groups (Fig. 6f), three models demonstrated excellent performance, each with AUC values greater than 0.8. These models were based on (a) PN, DMN, SMN, and Ins–DMN; (b) PN, DMN, and SMN; and (c) PN and DMN. In summary, these findings indicated that appropriate classification models displayed favourable generalisability for identifying patients with CM in an independent dataset. Moreover, the connectivity features, primarily from the PN and DMN, may be significant for characterising the neuropathology of CM. Additionally, receiver operating characteristic curves from each validation model are plotted using decisive values (Fig. 7). Notably, receiver operating characteristic curves using predicted labels are depicted in Fig. 6.

## Discussion

In this study, we utilised the connectivity measurements of resting-state neuromagnetic activities to identify network-based features that can be used to distinguish patients with CM or other pain disorders from HCs. These features were primarily obtained from oscillatory connectivity across different frequencies and extracted from intrinsic interactions within the PN, DMN, SMN, and Ins–DMN networks. The oscillatory and network patterns provided insights into distinct brain abnormalities associated with different pain disorders. Utilising a combination of distinct network features, the classification models demonstrated excellent performance for distinguishing patients with CM from HCs, exhibiting high accuracy for training and testing datasets. Furthermore, in validation tests, three classification models displayed strong performance for differentiating the CM group from all other groups (HC, CMFM, FM, CTTH, and EM). These findings suggest that resting-state MEG networks offer specificity and reliability for identifying patients with CM. In addition, connectivity features, primarily extracted from the PN and DMN, may play significant roles in characterising the neuropathology of CM.

### Characteristic brain network alterations in pain disorders

Aberrant resting-state FC is associated with distinct pain disorders. These alterations not only reflect symptoms [16] and severity [5, 15] but also have implications for treatment outcomes [29, 30]. Consistent with these previous observations, our results revealed decreased connectivity of resting-state MEG activities within brain networks across various oscillatory frequencies, indicating distinct patterns for different pain disorders. In particular, the CM and CMFM groups exhibited abnormal connectivity in the PN, DMN, SMN, and Ins–DMN; this finding aligns with those of previous studies [5, 9, 10, 13, 15, 29]. However, the network profiles for patients with CMFM have not been fully determined. This is the first study to demonstrate alterations in common networks for both disorders. These findings suggest that instead of a single unique network, a distributed and manifold set of brain networks is involved in the dysfunctional brain mechanisms in patients with CM or CMFM. This speculation aligns with the understanding that pain encompasses sensory, affective, and cognitive components. Consistent with the finding of a previous study [4], the patients with CTTH in this study exhibited abnormal connections only in the PN, indicating that CTTH is characterised as pain-related central neural dysfunction. These pieces of evidence highlight distinct central neuropathological mechanisms for tension-type headaches and migraines [4, 31]. Moreover, for FM, which is a widespread musculoskeletal pain disorder, FC within the PN was preserved, distinguishing FM from other headache disorders. Patients with FM display aberrant connections between the insula and DMN, which is associated with symptom severity [11]. These findings imply that connectivity patterns in the PN can serve as a crucial feature for differentiating FM from migraine. These patterns are associated with distinct underlying pathophysiologies [32] despite common alterations observed in the DMN, SMN, and Ins–DMN. Furthermore, as a disorder within the migraine spectrum, EM shares common network alterations with CM, except for the decreased connectivity in the PN and decreased gamma connectivity in the Ins–DMN. This finding aligns with that of a previous MEG study [5], which suggested that ACC connectivity within the PN was related to migraine chronification, indicating the presence of connectivity differences between EM and CM. Overall, the neuropathological features of CM may be specifically characterised by network patterns derived from the oscillatory connectivity of MEG resting-state activities.

From electrophysiological perspectives, oscillatory coupling reveals underlying abnormal neuronal mechanisms, particularly in neurological disorders [33]. The findings of this study revealed altered oscillatory connectivity in patients with pain disorders, indicating impairment in the flexible routing of information across brain areas at various oscillatory frequencies [34, 35]. Moreover, distinct oscillatory patterns within each network represent the characteristic features of different pain disorders. Pain results from the integration of nociceptive and contextual information and is mediated by feedforward and feedback processes in the brain [36], involving gamma and alpha/beta oscillations, respectively [37]. Thus, pain disorders can be identified based on the presence of altered oscillatory connectivity within networks, resulting from dysfunctional integration and mediation in the brain at various frequencies and deficits in different neurophysiological processes. In line with these notions, this study revealed distinct oscillatory characteristics of altered connectivity patterns among pain disorders. In particular, in CM and CMFM, beta and gamma synchrony was deteriorated in most brain networks. Moreover, altered theta connectivity and alpha connectivity in the PN and SMN were observed exclusively in CM. Aberrant gamma connectivity may be a common oscillatory feature among pain disorders. This observation is consistent with the fact that gamma oscillation, originally involved in encoding afferent sensory information over the sensory cortex, undergoes changes after long-lasting pain. Therefore, abnormal gamma oscillation begins to appear over brain areas responsible for emotional–motivational processing and dominates the processing and perception of pain [37, 38]. Moreover, abnormal gamma oscillation is associated with neurological and psychiatric symptoms resulting from thalamocortical dysrhythmia [39, 40]. Taken together, these findings indicate that the complex and unique oscillatory–spatial synchrony patterns of brain activity can serve as the signature for distinct pain disorders. Appropriate feature extractions from network-based oscillatory connectivity can facilitate the classification of pain disorders.

### Classification models for identifying patients with CM

In this study, we employed network-based features to establish a classification model for identifying patients with CM with high accuracy. The model using the validation data exhibited significant performance for discriminating migraine with comorbidities (CMFM), headache subtype (CTTH), musculoskeletal pain (FM), and migraine spectrum (EM), indicating the generalisability of this model for the differentiation of patients with CM from those with other pain disorders. Previous neuroimaging studies have identified patients with migraine by using ML algorithms. One our previous resting-state MEG study with SVM method [10], we obtain fine performance in distinguishing CM from EM or FM. However, this previous study had limitations concerning the specificity of the brain signatures obtained through node-node oscillatory connectivity in identifying CM from other primary headaches or chronic pain disorders. Moreover, it remains uncertain whether node-node connectivity within brain networks is capable of detecting alterations specific to CM. Particularly, network-based investigations can offer deeper insights into the functional relevance of sensory, affective, and cognitive aspects of cortical processes. To address these gaps, our current study included patients with different pain types (headache vs. musculoskeletal pain), headache subtypes (migraine vs. tension-type headache), migraine spectrum (chronic vs. episodic migraine), and comorbidity factors (CM with/without comorbid fibromyalgia) to examine the sensitivity/specificity of network-based oscillatory connectivity on patients with CM. Additionally, apart from the SVM method, we employed a variety of machine learning algorithms to assess the identification models. These included decision trees, discriminant analysis, naïve Bayes classifiers, and k-nearest neighbour. Furthermore, Shapley values were utilized to elucidate the contribution of each brain area to the classification model, offering valuable insights into potential targets for noninvasive migraine neuromodulation treatments.

In functional MRI studies, Schwedt and colleagues [41] established a CM classification model by using various parameters, including regional cortical thickness, cortical surface area, and volume, and the model achieved accuracies of 86.3% (CM [*n* = 15] vs. HC [*n* = 54]) and 84.2% (CM [*n* = 15] vs. EM [*n* = 51]). Another study using the functional connections of 33 seeded pain-related regions in the brain reported an accuracy of 86.1% for discriminating patients with migraine (*n* = 58) from that of HCs (*n* = 50) [42]. Moreover, the classification model using both functional and structural MRI features, including the amplitude of low-frequency fluctuations, regional homogeneity, regional functional correlation strength, and regional grey matter volume, displayed an accuracy of 83.67% for discriminating patients with migraine (*n* = 21) from HCs (*n* = 28) [43]. One study identified functional connections within the visual, default mode, sensorimotor, and frontoparietal networks and reported accuracies ranging from 84.2 to 91.4% for identifying patients with EM from HCs and 73.1% accuracy for distinguishing patients with EM from those with other chronic pain disorders (FM and chronic low back pain) [44]. Consistent with these findings, the present study asserts that functional network-based features can serve as the brain signatures of underlying pathophysiology and can be utilised for identifying patients with CM with high accuracy, particularly when the model incorporates oscillatory characteristics. An EEG study established a classification model by utilising evoked high-frequency oscillations in the somatosensory cortex, which achieved higher accuracies of 89.7% (HC [*n* = 15] vs. ictal migraine [*n* = 13]) and 88.7% (HC [*n* = 15] vs. interictal migraine [*n* = 29]) for identifying patients with migraine [45]. These findings imply that features derived from the oscillatory-spatial synchrony of brain activities facilitate the identification of patients with pain disorders. Although many migraine researches using functional MRI or EEG pointed to differentiate migraine patients from healthy controls, using MEG in combination with machine learning techniques offers several advantages. First, MEG provides millisecond-level temporal resolution, allowing researchers not only to capture rapid changes in neural activity but also to extract dynamics in the oscillatory activity. This is crucial for understanding the dynamic nature of migraine-related brain processes. Second, MEG directly measures the magnetic fields generated by neuronal electrical activity. This direct measurement offers a unique perspective on brain function, allowing for detailed analysis of neural oscillations and connectivity patterns associated with migraines. Third, MEG can be combined with advanced source localization techniques to identify specific brain regions responsible for migraine-related activity. This spatial information is valuable for understanding the precise neural mechanisms underlying migraines. Therefore, this study achieved outstanding results in identifying patients with CM. Notably, the classification model demonstrated exceptional accuracy in discerning pain type, headache subtype, migraine spectrum, and comorbidity factors. Furthermore, to ensure reliability and generalisability, the present study collected resting-state MEG data with network-based and oscillatory features from 350 participants. Consequently, this approach can identify individual differences in migraine patterns. This personalized approach can lead to tailored treatment plans, improving the overall management of migraines for patients.

### Pivotal networks in PN and DMN for CM

By employing feature selection (independent *t* test for CM vs. HC) and performing model validation (CM vs. other pain disorders), oscillatory connectivity in the PN and DMN emerged as crucial brain signatures for distinguishing patients with CM from those with other pain disorders and HCs, exhibiting excellent performance (sensitivity: 0.85, specificity: 0.75, accuracy: 75.7% and AUC: 0.8). Aberrant activities in the PN have been observed in individuals with pain disorders, which are characterised not only by cortical hyperexcitability and sensory cortex disinhibition [7] but also by dysfunctional network connections between brain areas [5]. These phenomena have been consistently observed in patients with CM and have even been used as the indicators of disease severity [7] or treatment outcomes [6]. Additionally, alterations in connectivity within the DMN have been identified in patients with CM [9, 13]. These abnormalities are associated with emotional and cognitive disorders, as revealed by the examination of the white matter microstructure [12], anatomical connections [46], and structural and functional brain connectivity [14]. These findings collectively suggest that incorporating features from both the PN and DMN can comprehensively and precisely represent the underlying pathophysiological mechanisms in patients with CM, including altered sensory, affective, and cognitive processes. In general, oscillatory- and network-specific signatures utilised in this model serve as potential targets for noninvasive treatment options for migraine neuromodulation through transcranial magnetic and direct current stimulation.

### Limitations

This study has several limitations. First, altered cortical networks were examined and characterised for pain disorders in this study. However, networks between subcortical areas (such as the brainstem, thalamus, and hypothalamus) or their connections to cortical areas remained unresolved due to the limitations of MEG in recording deep brain activities. Second, the identification of patients with CM by using this model can facilitate individualised clinical decisions. However, whether this model can reveal the severity and progression of diseases or aid in determining the outcomes of treatments or interventions remain unclear. Future studies with longitudinal designs are necessary to explore the further applications of these models. Third, this study investigated oscillatory connectivity within the 1–40 Hz band. Interestingly, prior research has indicated a connection between abnormal high gamma or high-frequency oscillations and migraine patients [47, 48]. The impacts of these high-frequency oscillations on identifying migraine patients merit further investigation. Forth, gender disparities in pain disorders were observed due to their prevalence. Research indicates that FM predominantly affects women (80–90% of cases) [49], while the prevalence of migraine is 8.6% for males and 17.0% for females, and tension-type headache (TTH) affects 23.4% of males and 27.1% of females [50]. The recruited patient population in this study reflected these gender distribution patterns. The classification models developed during training processes did not consider gender differences between HC and CM since no distinction between the two groups. However, during validation analysis, these models effectively distinguished between CM vs. FM and CM vs. CTTH (achieving accuracy > 75%) even gender distribution differences. Interestingly, accuracy of identification among patients did not reveal significant effect on the factor of gender. This finding underscores the need for further investigations. Finally, none of the participants had received any migraine preventive medication. This decision was intentional, aiming to control for medication effects. However, this deviates from typical treatment patterns observed in patients with migraine in the clinical setting. This precluded us from determining whether our model can be generalised to patients receiving such treatments.

## Conclusion

Oscillatory and network patterns provided insights into the distinct brain abnormalities associated with different pain disorders. Functional activities within the PN and DMN corresponded to altered and distributed neurophysiological processes in patients with CM, making them pivotal signatures in the model for identifying patients with CM. With satisfactory reliability and generalisability, this classification model can facilitate the objective and individualised diagnosis of migraine.

## Data Availability

Derived data supporting the findings of this study are available on request from the corresponding authors.

## Abbreviations

CM: Chronic migraine
PN: Pain-related network
DMN: Default mode network
SMN: Sensorimotor network
VN: Visual network
Ins-DMN: Insula to DMN
ML: Machine learning
MEG: Magnetoencephalography
FC: Functional connectivity
HCs: Healthy controls
CMFM: CM with comorbid fibromyalgia
FM: Fibromyalgia
CTTH: Chronic tension-type headache
EM: Episodic migraine
HADS: Hospital anxiety and depression scale
MIDAS: Migraine disability assessment
3D: Three-dimensional
EOG: Electrooculography
ECG: Electrocardiography
MNE: Minimum norm estimate
ROIs: Regions of interest
MI: Primary motor
SI: Primary somatosensory
SII: Secondary somatosensory
ACC: Anterior cingulate cortex
FDR: False detection of discovery
SVM: Support vector machine
AUC: Area under the curve
